# Differences in miRNA and mRNA Profile of Papillary Thyroid Cancer Variants

**DOI:** 10.1155/2016/1427042

**Published:** 2016-08-30

**Authors:** Tomasz Stokowy, Danuta Gawel, Bartosz Wojtas

**Affiliations:** ^1^Department of Clinical Science, University of Bergen, Postboks 7804, 5020 Bergen, Norway; ^2^Department of Automatic Control, Akademicka 16, 44-100 Gliwice, Poland; ^3^Department of Nuclear Medicine and Endocrine Oncology, M. Sklodowska-Curie Memorial Cancer Center and Institute of Oncology, Gliwice Branch Wybrzeze AK 15, 44-101 Gliwice, Poland; ^4^Laboratory of Molecular Neurobiology, Neurobiology Center, Nencki Institute of Experimental Biology, Warsaw, Poland

## Abstract

Papillary thyroid cancer (PTC) can be divided into classical variant of PTC (cPTC), follicular variant of PTC (fvPTC), and tall cell variant (tcPTC). These variants differ in their histopathology and cytology; however, their molecular background is not clearly understood. Our results shed some new light on papillary thyroid cancer biology as new direct miRNA-gene regulations are discovered. The Cancer Genome Atlas (TCGA) 466 thyroid cancer samples were studied in parallel datasets to discover potential miRNA-mRNA regulations. Additionally, miRNAs and genes differentiating PTC variants (cPTC, fvPTC, and tcPTC) were indicated. Putative miRNA regulatory pairs were discovered: hsa-miR-146b-5p with PHKB and IRAK1, hsa-miR-874-3p with ITGB4 characteristic for classic PTC samples, and hsa-miR-152-3p with TGFA characteristic for follicular variant PTC samples. MiRNA-mRNA regulations discovery opens a new perspective in understanding of PTC biology. Furthermore, our successful pipeline of miRNA-mRNA regulatory pathways discovery could serve as a universal tool to find new miRNA-mRNA regulations, also in different datasets.

## 1. Introduction

Papillary thyroid cancer (PTC) is the most frequent thyroid cancer (80% of cases) with the 10-year overall relative survival rate of 93% [1, 2]. The other differentiated thyroid cancer that originates from follicular thyroid cells, follicular thyroid cancer (FTC), is less frequent with incidence of around 10% [1]. Most of PTC tumors have good prognosis and are relatively easy to treat [3]. Presence of tall cells in PTC is also considered as a risk factor, especially if percentage of tall cells comprises more than 10% of tumor cells [4]. The most frequent histopathological subtypes of PTC are classical variant of PTC (cPTC) and follicular variant of PTC (fvPTC), which are different in histopathology, but they confer similar risk of aggressive outcome, which in case of both tumors is relatively low [5, 6]. On both molecular and morphological levels fvPTC shares similarities with cPTC and follicular tumors, namely, FTC and follicular thyroid cancer (FTA) [7]. Follicular variant of PTC, especially encapsulated fvPTC, shares clinicopathological features of cPTC and FTC [8]. On the other hand, on molecular level fvPTC can harbor a BRAF V600E mutation and PAX8/PPARG translocation [9, 10]. Follicular variant of PTC standing in the middle of two distinct tumor types creates diagnostic challenge and incorrect diagnosis possibility. Incorrect classifications of fvPTC-FTA could be problematic and dangerous for the patient [11].

Genomic alterations, like BRAF mutation or PAX8/PPARG translocation, are not distinct features of fvPTC, first one being characteristic for cPTC and the second one for FTC and FTA. Expression markers may be helpful to distinguish follicular variant from other entities and number of studies were performed with use of both gene expression and miRNA expression markers [12–15]. Studies on the expression markers were limited by sample size and therefore a larger sample cohort would be desirable to catch population diversity.

Over the last decade miRNA importance in thyroid pathology was intensively studied with first publication emerging in 2005 [16]. A number of important direct miRNA-mRNA regulations were described in PTC, as hsa-miR-155 downregulating APC (adenomatous polyposis coli), THRB (thyroid hormone receptor beta) regulation by hsa-miR-21 and hsa-miR-146a, or hsa-miR-146b-5p regulating SMAD4 (SMAD family member 4) [17–19]. Specific effect of hsa-miR-155 downregulating APC expression was an increase in cell viability and colony formation in vitro [19]. SMAD4 expression regulation by hsa-miR-146b-5p modulated TGF-*β* signal transduction [18]. Both hsa-miR-21 and hsa-miR-146a targeting THRB caused a tumor suppressive effect on PTC development [17]. MiRNA regulatory pathways will indisputably shed some new light on papillary thyroid cancer biology as new direct miRNA regulations will be discovered.

In most of the cases when new miRNA-gene regulatory pair is introduced it is unclear how the pair was selected. Most frequently bioinformatics analysis leading to selection of interesting miRNA regulatory pathway is poorly described, not reproducible or just missing. Some publications are very focused on finding regulation/regulations of one chosen gene [17] or gene regulation/regulations by one chosen miRNA [18]. Global analysis of thyroid cancer miRNA regulations was done in PTC and in follicular thyroid tumors (FTC, FTA) [20, 21]. Both experiments were performed using microarray platforms for both gene and miRNA expression analysis with small sample size for follicular thyroid tumors (total 24 samples) [20] and relatively large sample size for PTC samples (126 samples) [21]. One problem with expression measurement by hybridization method applied in microarrays is that very similar sequences will hybridize to the same probe on microarray, which is extremely important especially for miRNAs, which are small in size and have been proven to have multiple isoforms ranging in size and in sequence [22]. In our opinion high-throughput sequencing is a method more suitable for correct miRNA expression evaluation since it is more isoform specific and enables us to evaluate expression of each miRNA isoform.

Lately published repository of deep sequencing data comprises a large dataset of PTC samples. The Cancer Genome Atlas (TCGA) project enables us to perform large scale analysis with high number of samples, and experiments were performed with high-throughput sequencing, a method most suitable to study miRNA expression [23]. In parallel, the TCGA thyroid cancer dataset covers gene expression data studied by RNA-Seq for the same cohort of patients.

## 2. Material and Methods

### 2.1. Samples

We analyzed miRNA and mRNA expression profile of 466 thyroid cancer samples sequenced in The Cancer Genome Atlas (TCGA): http://cancergenome.nih.gov/. Data included 321 Thyroid Papillary Carcinoma, classical/usual; 99: Thyroid Papillary Carcinoma, follicular (≥99% follicular patterned); 35: Thyroid Papillary Carcinoma, tall cell (≥50% tall cell features); and 11: other thyroid tumor samples. All 466 samples were annotated as presented in Supplementary File 1 (in Supplementary Material available online at http://dx.doi.org/10.1155/2016/1427042), Sample Annotations. Out of all 500 TCGA repository thyroid cancer samples we extracted samples for which both mRNA and miRNA high-throughput sequencing data were available.

### 2.2. Analysis of miRNA High-Throughput Sequencing Data

Expression of 46681 unique miRNA isoforms was detected in the miRNA sequencing data in at least one sample of all available in TCGA thyroid samples. Each of the isoforms was described by reads per million miRNA mapped as described in the TCGA data portal. 4133 of those isoforms were detected in at least half of 466 studied samples. Only those 4133 were taken into further consideration to diminish effect of low abundance isoforms on the outcome of the expression analysis. Quantile normalization with R/Bioconductor preprocessCore library was performed. After normalization log_2_ transformation of data was carried out together with subtraction of batch effects. Batch effect removal was performed by subtraction of average expression from each of 16 batches for each single miRNA isoform and for mRNA data. Results of batch removal were observed in the Principal Components Analysis of data before removal (Supplementary Figure  1A) and after removal (Supplementary Figure  1B). miRNA isoform data obtained from above preprocessing steps were passed to integrative analysis.

### 2.3. Analysis of mRNA High-Throughput Sequencing Data

20531 genes were detected in the mRNA sequencing data. Normalized counts of sequences aligning to particular genes were extracted for all of 466 samples common for mRNA, miRNA, and clinical data. 17438 genes were expressed in at least half of the samples so they were considered in the further analysis. Similar to miRNA data preprocessing log_2_ transformation, quantile normalization and batch removal were performed. Results of batch removal for gene expression data were presented in Supplementary Figure  1C (before batch removal) and Supplementary Figure  1D (after removal).

### 2.4. Statistical Testing and Annotation

Median based fold changes (MBFC) were introduced to diminish outlier effects and considered significant when higher than 1.25 or lower than 0.8. *t*-test *p* values were considered as statistically significant when lower than 0.05. False discovery rates (FDR) were computed with reference to total number of miRNAs on the microarray to take into account multiple testing bias. To compute *t*-test and FDR only expression features with variance higher than 1st quartile (25%) of variance in analyzed dataset were considered. Annotations were prepared according to miRBase version 20 [24–27] (ftp://mirbase.org/pub/mirbase/CURRENT/genomes/hsa.gff3).

### 2.5. Integrative Analysis of miRNA and mRNA

Preprocessed expression data of miRNA (4,133 annotated) and mRNA (17438 annotated) were correlated with each other independently in 3 sample groups: cPTC (321 samples), fvPTC (99 samples), and tcPTC (35 samples). Spearman correlation coefficient was calculated with respective *p* value for each correlation using Hmisc R library [28]. For each one of sample groups (cPTC, fvPTC, and tcPTC) a number of 72,071,254 independent correlation values with their respective *p* values were calculated. Raw *p* values were adjusted with False Discovery Rate (FDR) method. Correlation coefficient threshold of < −0.6 was applied to filter the best inverse correlations. Subsequently best inverse correlations for all 3 samples sets (cPTC, fvPTC, and tcPTC) were tested with miRNA regulation prediction algorithms: miRanda and TargetScan [29, 30]. For this analysis purpose miRNA chromosomal coordinates annotations were transformed to miRNA IDs according to miRBase version 20 (ftp://mirbase.org/pub/mirbase/CURRENT/genomes/hsa.gff3, chromosomal coordinates annotations being still primary annotations). Both algorithms (miRanda and TargetScan) were applied to predict miRNA binding sites in 3′-UTR and 5′-UTR and coding sites of mRNAs. Correlation of <−0.6 from all 3 sample groups was considered for further testing when both applied prediction tools (miRanda and TargetScan) were independently predicting a putative regulation of miRNA for miRNA-mRNA pair in tested correlations. To test analysis pipeline utility the best putative regulations were tested with isoform specific method of miRNA-target prediction: TargetRank, which takes into account binding of the seed region of miRNA to target mRNA sequence [31]. Only best putative regulations that were predicted by both miRanda and TargetScan and had *r* coefficient value of correlation below −0.65 for cPTC (321 samples in correlation), below −0.7 for fvPTC (99 samples in correlation) and −0.8 for tcPTC (35 samples in correlation), were tested with TargetRank algorithm. For better explanation of our analysis pipeline appropriate block diagram was presented in Supplementary Figure  2.

## 3. Results

### 3.1. Molecular Difference between fvPTC and tcPTC

**Figure 1:**
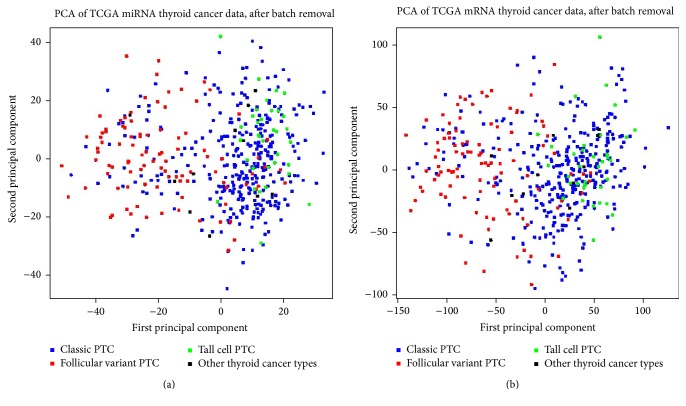
Principal Components Analysis of miRNA expression (a) and gene expression (b) data. fvPTC samples and tcPTC samples gather in 2 barely overlapping clusters (left and right parts of the plots, resp.). fvPTC and tcPTC difference represents the 1st principal component in both datasets; thus, it is the main source of miRNA and mRNA expression variability in the entire experiment.

Molecular difference between fvPTC and tcPTC was observed in the unsupervised analysis of expression data. For both miRNA and mRNA datasets we performed unsupervised Principal Components Analysis (PCA) and observed diverse clusters of samples (Figures 1(a) and 1(b)). 1552 miRNA isoforms and 8461 genes were differentiating fvPTC and tcPTC with FDR < 0.05. The most differentiating miRNA was hsa-miR-21-5p (FDR = 4.96*∗*10^−26^, MBFC = 0.35, downregulated in fvPTC) whereas the most differentiating gene was SFTPB (FDR = 3.20*∗*10^−31^, MBFC = 0.01, downregulated in fvPTC).

### 3.2. Follicular Variant of PTC and Its Molecular Difference from Classic PTC

**Figure 2:**
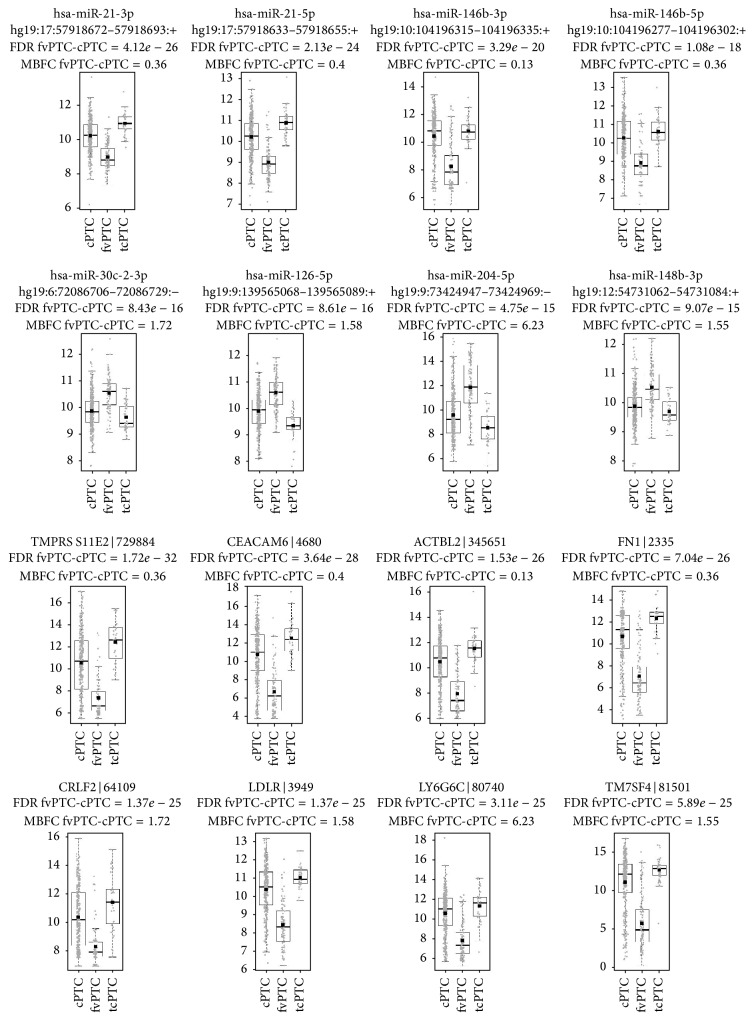
miRNAs and genes with the most differentially changed expression in classic PTC versus follicular variant PTC. Distributions of cPTC and fvPTC expression data overlap significantly; on the other hand, fvPTC and tcPTC distributions are relatively diverse. Top 8 miRNAs and genes were selected according to rules presented in Section 2, Statistical Testing and Annotation. miRNA data were annotated with hsa-miR number and hg19 isoform loci, whereas gene expression data were annotated with gene symbol and Gene ID.

We find fvPTC standing in between of two thyroid cancer types, PTC and FTC, and therefore we show differences of this variant contrary to cPTC based on TCGA data. Molecular difference between fvPTC and cPTC is of similar level to fvPTC and tcPTC difference, 1689 miRNA isoforms and 8721 genes differentiating with FDR < 0.05. The most differentiating miRNA is hsa-miR-21-3p (FDR = 4.12*∗*10^−26^, MBFC = 0.36, downregulated in fvPTC) and the most differentiating gene is TMPRSS11E2 (FDR = 1.72*∗*10^−32^, MBFC = 0.36, downregulated in fvPTC). Distributions of the most differentiating miRNAs and genes (fvPTC versus cPTC) are presented on the box plots in Figure 2. Top 8 miRNAs and genes with both significant FDR and significant MBFC were presented in the figure.

### 3.3. miRNA-mRNA Regulations in PTC

**Figure 3:**
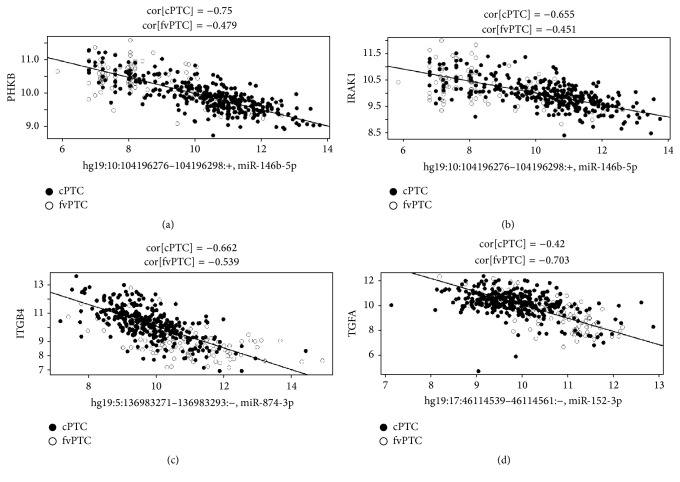
Putative miRNA-gene regulations. Pairs hsa-miR-146b-5p with PHKB (a), hsa-miR-146b-5p with IRAK1, (b) and hsa-miR-874-3p with ITGB4 (c) were selected from best inverse Spearman's correlations (below −0.65) within 321 cPTC samples and confirmed with miRanda, TargetScan, and TargetRank prediction algorithms. Pair hsa-miR-152-3p with TGFA (d) was selected from best inverse Spearman's correlations (below −0.70) within 99 fvPTC samples and confirmed with miRanda, TargetScan, and TargetRank algorithms. Gene expression values were plotted on *y*-axis whereas miRNA expression values on *x*-axis. Both cPTC (filled dots) and fvPTC (empty dots) samples are depicted. Lines on graphs represent regression lines (gene expression values ~ miRNA expression values) for cPTC samples ((a), (b), (c)) and regression line for fvPTC samples (d). Approximated correlation coefficients (*r*) are calculated for both cPTC and fvPTC samples and presented in the above graphs.

Correlation coefficient threshold of *r* < −0.6 applied resulted in a list of 3488 miRNA-mRNA correlation pairs for cPTC (321 samples), 9460 for fvPTC (99 samples), and 35028 for tcPTC (35 samples). Out of correlations below −0.6 threshold miRanda or TargetScan has predicted 370 independent putative miRNA regulations targeting 3′-UTR, 5′-UTR, or coding region of mRNA for cPTC samples, 1786 for fvPTC, and 10492 for tcPTC. CPTC had 97 putative miRNA regulations confirmed independently by two prediction tools (miRanda and TargetScan), fvPTC had 483, and tcPTC had 2628. In Supplementary Tables  1, 2, and 3, we presented putative miRNA regulations predicted independently by two prediction algorithms (miRanda and TargetScan). Calculated correlation coefficients from TGCA dataset below −0.65 for cPTC samples (321 samples correlated), below −0.70 for fvPTC (99 samples correlated), and below −0.80 for tcPTC (35 samples correlated) were considered for further testing. TargetRank algorithm tested on putative regulations with *r* < −0.65 for PTC, *r* < −0.70 for fvPTC, and *r* < −0.80 for tcPTC has confirmed 4 putative miRNA regulatory pairs: hsa-miR-146b-5p with PHKB and IRAK1; hsa-miR-874-3p with ITGB4 within cPTC samples (Table 1, Figure 3). Hsa-miR-152-3p with TGFA pair was confirmed within fvPTC samples (Table 1, Figure 3).

## 4. Discussion

We present in this work a successful pipeline of analysis for miRNA-mRNA regulation discovery in a large dataset of PTC samples from TCGA project. An important statement to make is that our analysis pipeline is designed only for miRNAs causing cleavage of target mRNAs, while it would rather fail in discovering or confirming miRNA triggered translation repression. A simple reason for that is that we correlate in our analysis mRNA expression with miRNA expression and we lack protein expression data to study translation repression.

Described miRNA-mRNA regulations were obtained with very stringent filtering criteria: best inverse correlation coefficient values (<−0.65 for cPTC, <−0.70 for fvPTC, and <−0.80 for tcPTC), additionally confirmed independently with 3 prediction algorithms (miRanda, TargetScan, and TargetRank). We provide extended tables with putative miRNA regulatory pairs with correlations below −0.60, confirmed by miRanda and TargetScan for all studied PTC histotypes in supplemental materials (Tables  S1, S2, and S3). Sample size of tcPTC set (35 samples) is a limiting factor in analysis and one should be cautious with drawing conclusions for this sample subgroup. Thus, main effort was done to compare fvPTC and cPTC sample sets.

Out of the best correlations (below −0.60) calculated for cPTC (3488 correlations), fvPTC (9460 correlations), and tcPTC (35028 correlations) 10–30% were confirmed by miRanda or TargetScan prediction algorithms. Both miRanda and TargetScan concordantly were predicting 3–7.5% from best correlations (below −0.60). Low percent of those confirmed miRNA regulations from all of the correlation results may seem surprising. On the other hand, large part of high correlations may simply represent coexpression or coregulation of miRNAs and genes. Putative regulation discovery should be well reinforced by suitable confirmation to avoid false positives, coming from biological processes that may also produce high correlation coefficient values.

MiRNAs most deregulated between cPTC and fvPTC were different isoforms of hsa-miR-21 and hsa-miR-146b, hsa-miR-30c-2-3p, hsa-miR-126-5p, hsa-miR-204-5p, and hsa-miR-148b-3p (Figure 2). Hsa-miR-21 was shown to be significantly deregulated in solid tumors [32]. Moreover, it was one of four miRNAs (with hsa-miR-222, hsa-miR-328, and hsa-miR-197) included in the classifier that reported sensitivity of 100% and specificity of 86% for a predictive accuracy of 90% in differentiating malignant from benign indeterminate lesions [33]. More importantly hsa-miR-21 and hsa-miR-146b along with 3 other miRNAs (181b, 221, and 222) were reported to be significantly upregulated in PTC when compared to both FAs and multinodular goiters (MNG), suggesting that both miRNAs may be characteristic for classical PTC and not for benign lesions (MNG, FA) [13]. In our work we report that both hsa-miR-146b and hsa-miR-21 isoforms are downregulated in fvPTC when compared to cPTC. Upregulation of other miRNAs on fvPTC when compared to cPTC (miRNA 30c-2-3p, 126-5p 204-5p, and 148b-3p) was to the best of our knowledge not reported yet.

We have presented the most significantly deregulated genes between cPTC and fvPTC: TMPRSS11, CEACAM6, ACTBL2, FN1, CRLF2, LDLR, LY6G6C, and TM7SF4 (Figure 2). Fibronectin 1 (FN1) is a well-described PTC marker [34, 35]. However, we should say that based on our results it is a marker of cPTC samples, as it was highly upregulated in cPTC samples. To the best of authors knowledge, remaining genes (TMPRSS11, CEACAM6, ACTBL2, CRLF2, LDLR, LY6G6C, and TM7SF4) were never reported in the context of thyroid neoplasia and according to our results they should be considered as differentiation markers between cPTC and fvPTC (Figure 2).

According to our integrative analysis we report four new regulations in PTC: hsa-miR-146b-5p regulating PHKB (phosphorylase kinase, beta), hsa-miR-146b-5p regulating IRAK1 (interleukin-1 receptor-associated kinase 1) and hsa-miR-874-3p regulating ITGB4 (integrin, beta 4) in cPTC samples, and hsa-miR-152-3p regulating TGFA (transforming growth factor, alpha) in fvPTC samples. TGFA/EGFR pair is one of the most tightly controlled ligand/receptor pairs in PTC samples [36]. In light of our results in a fvPTC histotype miRNA-152-3p may control TGFA expression and balance proliferation/differentiation signals of TGFA on thyroid cells (Figure 3). hsa-miR-152-3p regulation of TGFA expression is rather absent or antagonized by other regulation mechanisms in cPTC, correlation of this two RNAs expressions in cPTC samples is rather high (−0.42), and samples distribution is not suggesting a strong dependence of TGFA expression on hsa-miR-152-3p expression (Figure 2).

Integrin, beta 4 is involved in cell adhesion and motility [37, 38]. Putative regulation of ITGB4 expression by hsa-miR-874-3p was observed in cPTC samples and may be also present in fvPTC variant (Figure 3). Expression of ITGB4 is low in most of fvPTC samples and rather high in cPTC samples suggesting different patterns of regulation and/or additional regulation of ITGB4 expression (Figure 3). PTC variants are known to be different in their metastatic potential, as fvPTC more often gives distant metastases and cPTC is more frequently observed with local node metastases and soft tissue invasion noted during surgery [39]. Hsa-miR-874-3p was already reported to control cell invasiveness in non-small cell lung and gastric cancers [40, 41]. Regulation of ITGB4 may be a one aspect controlling PTC cells invasive potential and as presented on Figure 3 there are samples of PTC where miRNA-874-3p is significantly downregulating ITGB4 expression and there are samples that escape this regulation and have high ITGB4 expression.

Hsa-miR-146b-5p is very well described in a context of thyroid cells biology and neoplastic transformation [15, 18, 42]. In cPTC it may regulate PHKB and IRAK1 genes expression, and both regulations are most likely absent or antagonist by other regulatory processes in fvPTC (Figure 3). What is striking about both regulatory pairs is that regulation seems to be present only when hsa-miR-146b-5p expression is relatively high (approximately more than 8, Figure 3), suggesting a threshold of miRNA expression that is necessary to trigger the regulation. Most of fvPTC samples have lower hsa-miR-146b-5p expression and that is why they escape this regulation by miRNA. PHKB is a gene encoding enzyme involved in carbohydrates metabolism [43]. Hsa-miR-146b-5p may control carbohydrate metabolism in cPTC, as energy supply is crucial for cancer cell development. Second putative regulation exerted by hsa-miR-146b-5p is IRAK1 regulation that is in fact a confirmed and validated miRNA regulation [44–46], reported also by miRTarBase, database of experimentally validated miRNA-target interactions [47]. In fact a closely related hsa-miR-146a was already suggested to regulate IRAK1 in PTC [48]. We find highly concordant results regarding hsa-miR-146b-IRAK1 regulation as an indirect proof that our pipeline of analysis is correctly predicting real miRNA regulations in PTC. IRAK1 has been reported to have pleiotropic effect on cell biology, so it is hard to speculate what functional effect hsa-miR-146b-5p regulation of IRAK1 can produce. As IRAK1 is activated by interleukin-1 it is very likely that miRNA 146b controlling IRAK1 activity has a modulating effect on interactions with inflammatory cells infiltrating thyroid parenchyma.

## 5. Conclusion

A successful pipeline of miRNA-mRNA regulatory pathways discovery was proposed and applied to 466 miRNA and mRNA high-throughput sequenced samples. 8 human miRNA expression markers differentiating between conventional and follicular variants of PTC were proposed: 21-3p, 21-5p, 146b-3p, 146b-5p, 30c-2-3p, 126-5p 204-5p, and 148b-3p. Similarly 8 gene expression markers differentiating between conventional and follicular variants of PTC were observed: TMPRSS11, CEACAM6, ACTBL2, FN1, CRLF2, LDLR, LY6G6C, and TM7SF4. Four putative miRNA regulatory pairs were discovered: hsa-miR-146b-5p with PHKB and IRAK1, hsa-miR-874-3p with ITGB4 characteristic for cPTC samples, and hsa-miR-152-3p with TGFA characteristic for fvPTC samples. Our computational analysis proposed a number of miRNA-mRNA interactions acting in PTC. Results should be interpreted carefully and need further experimental verification in the light of currently available evidence.

## Supplementary Material

Supplementary File 1. Figure illustrates clustering of data due to batch effects present in TCGA miRNA and mRNA datasets (A, C). Samples sequenced in one batch are marked with the same color. After batch removal procedure clustering effects are not observed (B, D). Supplementary File 2. Figure illustrates analysis pipeline for 2 datasets - 466 samples. It provides overview how the study was designed.

## Figures and Tables

**Table 1:** Selection of 10 top scoring putative miRNA regulations in PTC, fvPTC, and tcPTC. Selected Spearman correlations of mRNAs and miRNAs were calculated independently in cPTC (321 samples), fvPTC (99 samples), and tcPTC (35 samples) datasets. All shown correlations were predicted by two used prediction tools: miRanda and TargetScan as putative miRNA regulations. Top 10 from each type of thyroid cancer (cPTC, fvPTC, and tcPTC) were ranked by correlation coefficient value and 10 lowest correlations are shown for each dataset (cPTC, fvPTC, and tcPTC). In columns from left, “regulation name,” assigned name of correlation; “correlation *r*,” correlation coefficient value (Spearman); “Correlation FDR,” DR corrected *p* value of correlation; “mature miRNA,” mature miRNA name; “confirmed by TargetRank,” stating that if regulation is confirmed by TargetRank (rank and score in the parenthesis); “gene symbol,” HGNC symbol of gene in correlation; and “gene name,” HGNC official full name.

Regulation name	Correlation *r*	Correlation FDR	Mature miR	Confirmed by TargetRank	Gene symbol	Gene name
PTC_1	−0.750	<1*e* − 12	hsa-miR-146b-5p	YES (19, 0.48)	PHKB	Phosphorylase kinase, beta
PTC_2	−0.681	<1*e* − 12	hsa-miR-146b-5p	NO	TMEM164	Transmembrane protein 164
PTC_3	−0.675	<1*e* − 12	hsa-miR-204-5p	NO	LAD1	Ladinin 1
PTC_4	−0.670	<1*e* − 12	hsa-miR-21-5p	NO	BTBD11	BTB (POZ) domain containing 11
PTC_5	−0.662	<1*e* − 12	hsa-miR-874-3p	YES (36, 0.39)	ITGB4	Integrin, beta 4
PTC_6	−0.657	<1*e* − 12	hsa-miR-204-5p	NO	ERBB3	v-erb-b2 avian erythroblastic leukemia viral oncogene homolog 3
PTC_7	−0.657	<1*e* − 12	hsa-miR-30c-2-3p	NO	EHBP1L1	EH domain binding protein 1-like 1
PTC_8	−0.655	<1*e* − 12	hsa-miR-146b-5p	YES (44, 0.41)	IRAK1	Interleukin-1 receptor-associated kinase 1
PTC_9	−0.655	<1*e* − 12	hsa-miR-30a-3p	NO	TNFSF11	Tumor necrosis factor (ligand) superfamily, member 11
PTC_10	−0.653	<1*e* − 12	hsa-miR-30c-2-3p	NO	RAP2B	RAP2B, member of RAS oncogene family
PTC_11	−0.653	<1*e* − 12	hsa-miR-146b-5p	NO	DNTT	DNA nucleotidylexotransferase
PTC_12	−0.653	<1*e* − 12	hsa-miR-146b-5p	NO	FHOD3	Formin homology 2 domain containing 3
PTC_13	−0.652	<1*e* − 12	hsa-miR-204-5p	NO	POU2F3	POU class 2 homeobox 3
fvPTC_1	−0.786	<1*e* − 12	hsa-miR-874-3p	NO	LASP1	LIM and SH3 protein 1
fvPTC_2	−0.729	<1*e* − 12	hsa-miR-484	NO	MET	met protooncogene
fvPTC_3	−0.724	<1*e* − 12	hsa-miR-152-3p	NO	LIPH	Lipase, member H
fvPTC_4	−0.714	<1*e* − 12	hsa-miR-874-3p	NO	SHF	Src homology 2 domain containing F
fvPTC_5	−0.714	<1*e* − 12	hsa-miR-874-3p	NO	LMNA	Lamin A/C
fvPTC_6	−0.709	3.89*e* – 12	hsa-miR-152-3p	NO	QSOX1	Quiescin Q6 sulfhydryl oxidase 1
fvPTC_7	−0.708	3.89*e* − 12	hsa-miR-152-3p	NO	PRR15	Proline rich 15
fvPTC_8	−0.707	6.83*e* − 12	hsa-miR-148b-3p	NO	CD276	CD276 molecule
fvPTC_9	−0.706	6.83*e* − 12	hsa-miR-874-3p	NO	PTK7	Protein tyrosine kinase 7
fvPTC_10	−0.704	6.83*e* − 12	hsa-miR-152-3p	NO	CORO2A	Coronin, actin binding protein, 2A
fvPTC_11	−0.704	6.83*e* − 12	hsa-miR-874-3p	NO	GNAI2	Guanine nucleotide binding protein (G protein), alpha inhibiting activity polypeptide 2
fvPTC_12	−0.703	6.83*e* − 12	hsa-miR-152-3p	YES (79, 0.38)	TGFA	Transforming growth factor, alpha
tcPTC_1	−0.874	1.02*e* − 05	hsa-miR-342-5p	NO	KCNG3	Potassium voltage-gated channel, subfamily G, member 3
tcPTC_2	−0.833	0.00018	hsa-miR-7-2-3p	NO	SDC3	Syndecan 3
tcPTC_3	−0.831	0.00020	hsa-miR-454-3p	NO	TBX10	T-box 10
