# Supplementary material for: Differences in miRNA and mRNA Profile of Papillary Thyroid Cancer Variants
Source: Int J Endocrinol. 2016 Aug 30;2016:1427042. doi: 10.1155/2016/1427042 (PMC5021476; doi:10.1155/2016/1427042)
Supplement: Supplementary file 1 — Figure illustrates clustering of data due to batch effects present in TCGA miRNA and mRNA datasets (A, C). Samples sequenced in one batch are marked with the same color. After batch removal procedure clustering effects are not observed (B, D). Supplementary File 2. Figure illustrates analysis pipeline for 2 datasets - 466 samples. It provides overview how the study was designed. [file 1427042.f1.zip › supl_final.pdf]

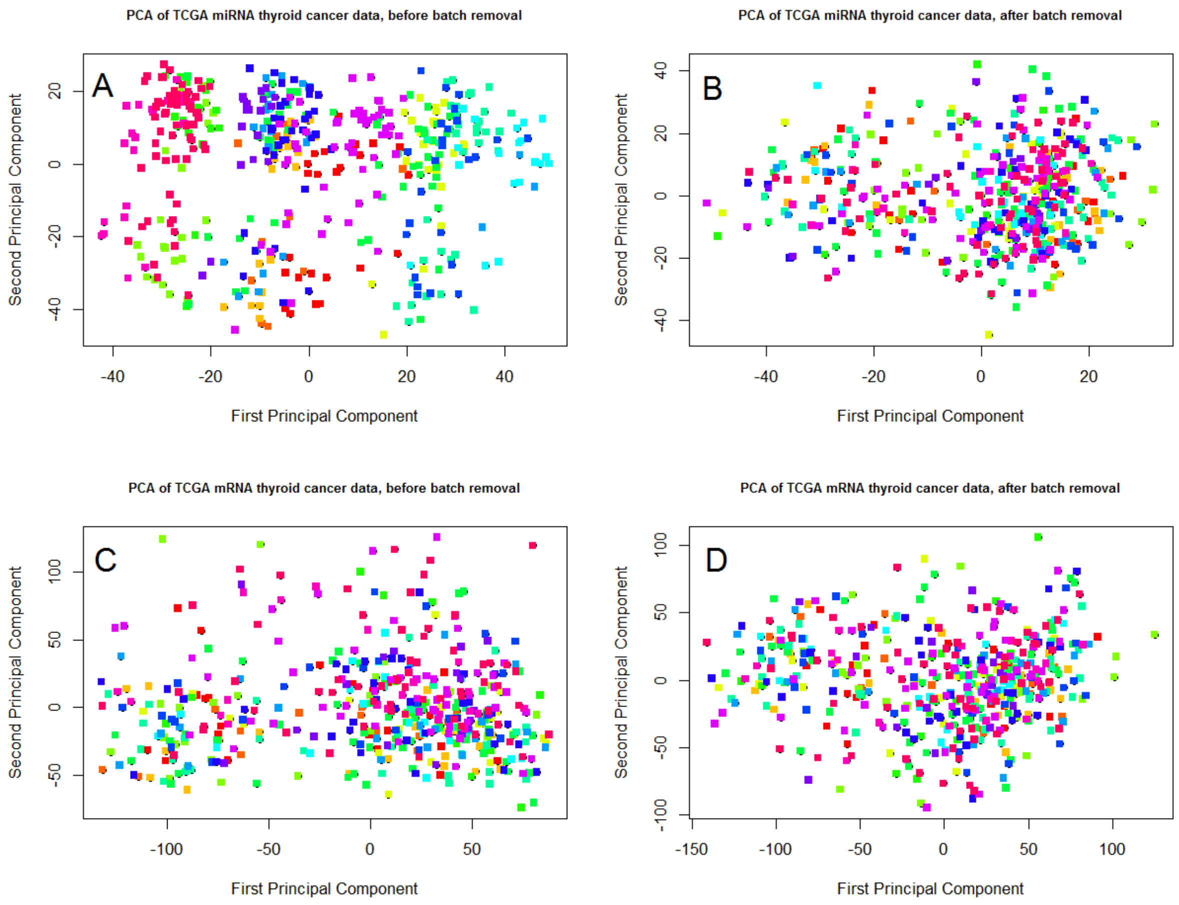

Figure S1.

Principal components analysis of miRNA (A,B) and mRNA (C,D) thyroid cancer TCGA data. Raw data (A, C) carry batch information induced by technical factor of sequencing series of samples. Particular rainbow colors represent 16 different batches annotated in the TCGA portal and Supplementary file 1. Batch effect is mainly visible in the miRNA data (A) as vertical clusters. Data after batch removal (described in the methods section) do not present any batch related clustering (B,D).

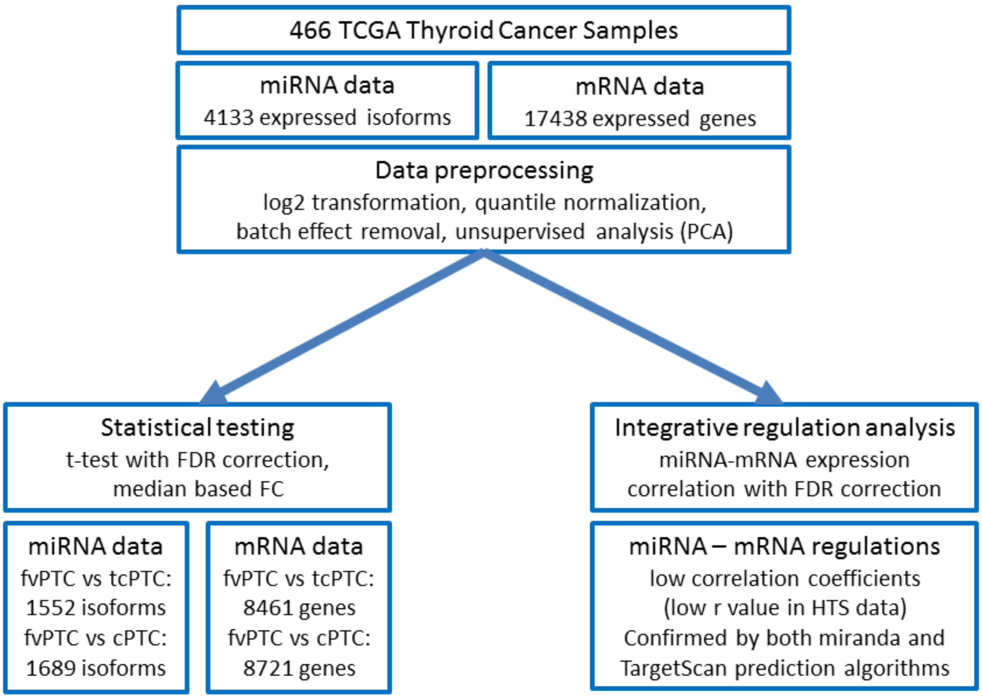

Figure S2

Scheme of analysis pipeline.

Table S1.

Putative miRNA regulations found in cPTC miRNA – mRNA expressions correlations results. All presented correlation pairs had r coefficient below -0.6 and all were confirmed by two prediction algorithms (miRanda, TargetScan). In columns following data are presented: miRNA position – chromosomal position of miRNA reads from high throughput sequencing, gene id – gene annotation from TCGA dataset, correlation – r coefficient value for presented correlation in cPTC samples (321), p-value – raw p-value for correlation, FDR – false discovery adjusted p-value, mature mir – mature miRNA name for annotated miRNA position, ensembl – ensembl id of gene from correlation pair, p3 miranda – miRNA regulation was predicted in 3'-UTR of gene for that correlation by miRanda algorithm (1) or not (0), p5 miranda – miRNA regulation was predicted in 5'-UTR of gene for that correlation by miRanda algorithm (1) or not (0), coding miranda – miRNA regulation was predicted in coding region of gene for that correlation by miRanda algorithm (1) or not (0), p3 targetscan – miRNA

regulation was predicted in 3'-UTR of gene for that correlation by TargetScan algorithm (1) or not (0), p5 targetscan – miRNA regulation was predicted in 5'-UTR of gene for that correlation by TargetScan algorithm (1) or not (0), coding targetscan – miRNA regulation was predicted in coding region of gene for that correlation by TargetScan algorithm (1) or not (0), correlation in fvPTC – Spearman's correlation r value for fvPTC samples (99)

Table S2.

Putative miRNA regulations found in fvPTC miRNA – mRNA expressions correlations results. All presented correlation pairs had r coefficient below -0.6 and all were confirmed by two prediction algorithms (miRanda, TargetScan). In columns following data are presented: miR position – chromosomal position of miRNA reads from high throughput sequencing, gene id – gene annotation from TCGA dataset, correlation – r coefficient value for presented correlation in fvPTC samples (99), p-value – raw p-value for correlation, FDR – false discovery rate adjusted p-value, mature mir – mature miRNA name for annotated miRNA position, ensembl – ensembl id of gene from correlation pair, p3 miranda – miRNA regulation was predicted in 3'-UTR of gene for that correlation by miRanda algorithm (1) or not (0), p5 miranda – miRNA regulation was predicted in 5'-UTR of gene for that correlation by miRanda algorithm (1) or not (0), coding miranda – miRNA regulation was predicted in coding region of gene for that correlation by miRanda algorithm (1) or not (0), p3 targetscan – miRNA regulation was predicted in 3'-UTR of gene for that correlation by TargetScan algorithm (1) or not (0), p5 targetscan – miRNA regulation was predicted in 5'-UTR of gene for that correlation by TargetScan algorithm (1) or not (0), coding targetscan – miRNA regulation was predicted in coding region of gene for that correlation by TargetScan algorithm (1) or not (0), correlation in cPTC – Spearman's correlation r value for cPTC samples (321)

Table S3.

Putative miRNA regulations found in tcPTC miRNA – mRNA expressions correlations results. All presented correlation pairs had r coefficient below -0.6 and all were confirmed by two prediction

algorithms (miRanda, TargetScan). In columns following data are presented: miR position – chromosomal position of miRNA reads from high throughput sequencing, gene id – gene annotation from TCGA dataset, correlation – r coefficient value for presented correlation in tcPTC samples (35), p-value – raw p-value for correlation, FDR – false discovery rate adjusted p-value, mature mir – mature miRNA name for annotated miRNA position, ensembl – ensembl id of gene from correlation pair, p3 miranda – miRNA regulation was predicted in 3'-UTR of gene for that correlation by miRanda algorithm (1) or not (0), p5 miranda – miRNA regulation was predicted in 5'-UTR of gene for that correlation by miRanda algorithm (1) or not (0), coding miranda – miRNA regulation was predicted in coding region of gene for that correlation by miRanda algorithm (1) or not (0), p3 targetscan – miRNA regulation was predicted in 3'-UTR of gene for that correlation by TargetScan algorithm (1) or not (0), p5 targetscan – miRNA regulation was predicted in 5'-UTR of gene for that correlation by TargetScan algorithm (1) or not (0), coding targetscan – miRNA regulation was predicted in coding region of gene for that correlation by TargetScan algorithm (1) or not (0), correlation in cPTC – Spearman's correlation r value for cPTC samples (321)
